# Utilization of primary health care services among Syrian refugee and Lebanese women targeted by the ICRC program in Lebanon: a cross-sectional study

**DOI:** 10.1186/s13031-019-0190-4

**Published:** 2019-03-15

**Authors:** Claudia Truppa, Enrica Leresche, Arlan F. Fuller, Ariana S. Marnicio, Josyann Abisaab, Nicole El Hayek, Carla Zmeter, Warda S. Toma, Hilda Harb, Randa S. Hamadeh, Jennifer Leaning

**Affiliations:** 1 The International Committee of the Red Cross, Beirut Delegation, Beirut, Lebanon; 2 Harvard François Xavier Bagnoud Center for Health and Human Rights, Boston, USA; 3 Department of Paediatrics, The University of British Columbia, Vancouver, Canada; 4 Lebanese Ministry of Public Health, Beirut, Lebanon

**Keywords:** Primary health care, Health services, Maternal and Child health, Lebanon, Syrian refugees, Host community, Humanitarian assistance, Mixed methods study

## Abstract

**Background:**

The Syrian crisis has put tremendous strain on the Lebanese health system, particularly in the historically underserved border region. The ICRC Primary Health Care program has focused on refugee and host communities in these areas. This study objectives were: 1) to determine whether the ICRC program was reaching the most vulnerable populations; 2) to understand the key perceived health needs in the catchment areas of the ICRC supported facilities; and 3) to identify barriers to utilization of health care services.

**Methods:**

Between July and September 2017 we conducted two cross-sectional studies - one randomized household survey and one clinic-based - in the catchment areas of three ICRC-supported facilities, targeting women of reproductive age and caretakers of children under five. Differences between groups were analysed with t-test or chi-squared test.

**Results:**

In the household survey, similar socio-demographic profiles were observed between Syrian refugee women and vulnerable Lebanese hosts. With regard to the study objectives:

1. The most vulnerable populations were those seen in the ICRC-supported facilities.
2. For both populations, the most common reasons for seeking care were non-communicable diseases (40.6%) and sexual and reproductive health issues (28.6%). Yet the people reaching the ICRC supported facilities were more likely to seek care for communicable diseases affecting their children (37.8%), rather than for the most common reasons expressed in the household survey.
3. In the catchment areas, reported gaps included low immunization coverage and low levels of antenatal care and family planning both for Syrian and Lebanese. Dental care also emerged as an issue. Out of pocket expenditures was reported as a critical barrier for utilization of primary health care services for both populations, while the most important barrier for utilization of ICRC-supported services was lack of awareness.

**Conclusions:**

Despite the ICRC reaching the most vulnerable Syrian and Lebanese communities, the population-based survey revealed that important gaps exist in terms of utilization of health care services among women of reproductive age and their children. A stronger outreach component is needed to address lack of awareness. Innovative solutions are also needed to address cost barriers at the levels of both facility and individual user.

## Background

As the country with the highest per capita ratio of refugees in the world [1], Lebanon is now heavily burdened by the Syrian crisis. In December 2018, the Government of Lebanon estimated hosting 1.5 million Syrian refugees, of which around 1 million are registered [2]. More than half of them are women, children and adolescents, currently struggling to get essential services [3]. Because of settlement patterns, the demographic pressure introduced by the refugees disproportionately affects the poorest and most underserved areas of Lebanon, leading to increasing socio-economic vulnerability of both populations [4]. This trend contributes to an increase in social tensions and competition for access to basic services and healthcare services, in particular in underserved areas [5, 6].

The Lebanese healthcare system is highly privatized, based on fee for service in both the public and private sector. In this context, the initial humanitarian health response to the Syrian crisis in Lebanon focused on subsidizing and financing health care services according to the established payment mechanisms [3]. The Ministry of Public Health (MoPH), supported by the World Health Organization (WHO) and the United Nations High Commissioner for Refugees (UNHCR), coordinated international and national Non-Governmental Organizations (NGOs) to ensure the widest possible coverage of services. Additional support for these services was granted by UNICEF, the World Bank and the European Union, in a joint effort to strengthen and expand the availability of basic primary health care services within the existing Primary Health Care (PHC) network of 223 health facilities operating all across the Lebanese territory. Out of these, 67% are affiliated with NGOs. Despite such efforts, however, the humanitarian health response has been fragmented and often inefficient [7]. Factors include lack of awareness among the populations residing in the most underserved areas of Lebanon of the availability of supported services [8] and challenges in ensuring cost control in a dominant privatized health system operating outside the MoPH network.

In response to the impact of the Syrian crisis on the Lebanese health system, the International Committee of the Red Cross (ICRC) has established since 2014 a PHC support program, structured as support to ten existing health facilities included in the above mentioned PHC network. The PHC support focuses on hard to reach areas overburdened by a high ratio of refugees and competition for basic services. The main objective of the program is to ensure access to essential preventative and curative health care services for the most vulnerable populations residing in catchment areas heavily affected by the Syrian crisis.

After 3 years of program implementation, in 2017 the ICRC conducted a study in partnership with the MoPH and the Ministry of Social Affairs (MoSA) and with the technical support of the Harvard François-Xavier Bagnoud (FXB) Centre for Health and Human Rights to evaluate the impact of its support program, particularly in relation to utilization of health services on the part of mothers and their children. The research objectives of the study were: 1) to determine whether the ICRC program was reaching the most vulnerable populations in catchment areas of ICRC supported facilities; 2) to understand the key perceived health needs among women of reproductive age and caretakers of children in the same areas; and 3) to identify barriers to utilization of health care services. The operational objective of this study was to contribute to evidence-based programming in ICRC health interventions in protracted crisis settings.

## Methods

We conducted a mixed-methods study between July and September 2017.

The quantitative component consisted of two cross-sectional studies, one household-based and another clinic-based. For both surveys, the study population included women of reproductive age between 18 and 50 years and caretakers of children less than 18 years of age as self-respondents – both women and men regardless of their age.

The household-based study was conducted within the population living in the catchment areas (defined as cadasters within a 7 km radius from the health facility) of ICRC-supported facilities in three Governorates: Beqaa, Nabatieh, and Akkar. The three facilities (out of ten) were selected for three reasons: 1) the high vulnerability of residents in these areas according to the UNHCR vulnerability classification; 2) the diversity in terms of funding bodies and services available; and 3) the differences in grades and types of ICRC support (Table 1). The exact location and name of the facilities cannot be provided in order to preserve the anonymity of the informants.

**Table 1:** Characteristics of the ICRC supported facilities included in the study

Characteristic	Beqaa	Akkar	Nabatieh
Ownership	Syrian NGO	MOSA	Private faith-based charity
Licensed by MOPH	Yes	N/A	Yes
Area	Urban + rural	Rural	Rural
Competing health facilities	+++	+	–
HR available	+++	–	++
Average number consultations per month^a^	~ 3600	~ 180	~ 1400
ICRC support (at the time of the survey)	• Supplies • Regular monitoring • Mental Health and Psycho-Social Support (MHPSS) • Community health	• Supplies • Regular monitoring • MHPSS	• Supplies • Regular monitoring

^a^ Based on ICRC institutional monitoring and reporting tool data (Medical Activity Database)

The household survey included a two-stage cluster-based population sample of all people living within the catchment areas, including registered and unregistered Syrian refugees, Lebanese in host communities, Palestinian refugees from Lebanon and from Syria, and any housekeepers or laborers from other nations residing in these areas. This method allowed for Syrian refugees within the catchment area to be sampled with equal likelihood, regardless of UNHCR registration status. The ICRC GIS officer obtained UNOSAT satellite images of the catchment areas of the three ICRC-supported facilities. The location of informal tented settlements (ITSs) was obtained from UNHCR and added as a second layer to the satellite images using ArcMap. The primary sampling units were the clusters selected through simple random sampling within each catchment area. The secondary sampling units were households selected by simple random sampling among all structures (buildings or ITSs) within one cluster.

The sample size calculated for the household survey was determined for the main outcome under study (attendance at an ICRC supported facility), assuming a conservative proportion of 0.50, with a significance level of 5%. Based on population data for the selected cadasters (administrative units) within each of the catchment areas derived from the Lebanese Government’s Central Administration of Statistics (CAS) and the UNHCR data portal for Syrian refugees, the required sample sizes computed for each catchment area were between 374 and 383 participants. To account for any errors in data collection, the sample size was increased to 400 participants from each area.

Ethical approval was obtained from the Institutional Review Board (IRB) of Harvard University and at the national level from the Lebanese MoPH and MoSA. At each site, local authorities, including heads of municipalities and military intelligence, were informed of the objectives and methodology of the study. Teams of interviewers (mainly Lebanese Red Cross volunteers) who underwent 2-day standardized training administered the questionnaire. All interviewers were trained in research ethics principles and instructed to read to all eligible participants an information sheet about the study and to request oral informed consent before proceeding to the interview.

The clinic-based study was conducted in the three ICRC-supported facilities, with convenience sampling from patients receiving services at the clinics during the days in which the interviewer was attending the clinics. The interviewer approached patients after their appointment was completed and provided information about the study objectives, with an invitation to the patient to agree to participate. The clinic study was also conducted in a fourth facility supported by the ICRC in Hermel Governorate, where, due to security issues for site access at the time the study was conducted, it was not possible to conduct the household survey.

The administration of two surveys (one at population level and the other at facility level) allowed comparison between those who live in the area and those who visit the facility along key parameters - perceived health needs, reported health seeking behaviors, and encountered barriers to access essential services.

The household and clinic survey instruments were designed by the study team in English, translated into Arabic, and back-translated into English for validation. Both versions were piloted and refined before starting data collection. Survey instruments were designed using the Qualtrics software package and collected offline using tablets.

Data from the household and clinic surveys were extracted from Qualtrics and imported to SAS (version 9.3) for analysis. For continuous measures, a two-sided T-test was used to compare means between two groups. Association between categorical variables was assessed with chi-squared test. An alpha level of 0.05 was applied throughout to denote statistical significance.

The qualitative component of the study consisted in 18 focus group discussions of approximately seven to 12 people each, conducted between July and August 2017 by a trained qualitative researcher from the Harvard FXB Center who was fluent in Arabic. The results will be presented in a separate paper.

In this paper, we report the main findings of the quantitative component of our mixed method study.

## Results

In the household survey, out of 1479 households approached, a total of 1214 agreed to participate in the study, corresponding to an overall response rate of 82%. The final study population included 1179 participants (excluding 35 of other nationalities with numbers too limited to be representative). Among the respondents included in the final analysis, 59.37% (700/1179) were Lebanese and 40.63% (479/1179) were Syrian refugees. This ratio corresponded closely to the one derived from UNHCR estimates [2].

For the clinic survey, 208 surveys were collected, and all participants approached agreed to take part in the study. The final study population was 201 participants (excluding seven respondents of other nationalities). The majority (177/201, 88.06%) of respondents were Syrian refugees, while 11.94% (24/201) were Lebanese, the ratio indicating relative under-reporting of Lebanese compared to estimates from the Government of Lebanon [3].

### Vulnerability profile of targeted beneficiaries versus reached beneficiaries

In both surveys, the Lebanese population was significantly older, lived in households of smaller size and had slightly fewer children than Syrian refugees. The Lebanese participants also reported a slightly higher educational level and greater access to employment opportunities (Table 2).

**Table 2:** Socio-demographic characteristics of the 1,179 participants in the household survey and the 201 participants in the clinic survey

	Household Survey			Clinic Survey		
	Lebanese (%)	Syrian refugees (%)	Total (%)	Lebanese (%)	Syrian refugees (%)	Total (%)
Total Respondents	700 (59.37)	479 (40.63)	1179	24 (11.94)	177 (88.06)	201
Age (mean)	36.85	32.38	35.02	35.29	31.31	31.79
Gender						
Men	34 (4.89)	30 (6.29)	64 (5.41)	3 (12.50)	12 (6.78)	15 (7.46)
Women	661 (95.11)	447 (93.71)	1113 (94.00)	12 (87.50)	165 (93.22)	186 (92.54)
Household Composition						
Children (mean)	2.95	3.20	3.05	2.64	3.48	3.38
Pregnancies (mean)	3.70	4.06	3.85	3.52	4.70	4.57
Household Size (mean)	4.95	5.27	5.08			
Education						
Never attended school	81 (11.79)	75 (16.20)	156 (13.57)	.	12 (6.78)	12 (5.97)
Primary school	287 (41.78)	241 (52.05)	528 (45.91)	14 (58.33)	130 (73.45)	144 (71.64)
Secondary school	163 (23.73)	107 (23.11)	270 (23.48)	9 (37.50)	22 (12.43)	31 (15.42)
Technical school	32 (4.66)	5 (1.08)	37 (3.22)	.	6 (3.39)	6 (2.99)
College	22 (3.20)	10 (2.16)	32 (2.78)	1 (4.17)	6 (3.39)	7 (3.48)
Graduate school	95 (13.83)	17 (3.67)	112 (9.74)	.	1 (0.56)	1 (0.50)
Occupation						
Homemaker	504 (76.25)	347 (78.86)	851 (77.29)	9 (37.50)	93 (52.54)	102 (50.75)
Manual labor	34 (5.14)	47 (10.68)	81 (7.36)	5 (20.83)	32 (18.08)	37 (18.41)
Skilled labor	95 (14.37)	6 (1.36)	101 (9.17)	3 (12.50)	6 (3.39)	9 (4.48)
Unemployed	28 (4.24)	40 (9.09)	68 (6.18)	3 (12.50)	25 (14.12)	28 (13.93)
Marital status						
Married	608 (87.23)	431 (89.98)	1039 (88.35)	23 (95.83)	163 (92.09)	186 (92.54)
Single	52 (7.46)	12 (2.51)	64 (5.44)	1 (4.17)	2 (1.13)	3 (1.49)
Widowed	24 (3.44)	25 (5.22)	49 (4.17)	.	10 (5.65)	10 (4.98)
Divorced	11 (1.58)	4 (0.84)	15 (1.28)	.	1 (0.56)	1 (0.50)
Source of Income						
Work	547 (79.62)	154 (33.26)	701 (60.96)	23 (95.83)	137 (77.40)	160 (79.60)
Family money	26 (3.78)	8 (1.73)	34 (2.96)	2 (8.33)	4 (2.26)	6 (2.99)
Cash Assistance	6 (0.87)	20 (4.32)	26 (2.26)	.	4 (2.26)	4 (1.99)
Vouchers	6 (0.87)	56 (12.10)	62 (5.39)	.	83 (46.89)	83 (41.29)
E-cards	2 (0.29)	77 (16.63)	79 (6.87)	.	59 (33.33)	59 (29.35)
Charity	17 (2.47)	83 (17.93)	100 (8.70)	.	35 (19.77)	35 (17.41%)

The proportion of Syrian refugees attending ICRC supported health facilities was higher than that detected at population level (88.1% versus 40.6%, respectively), and differed from the general population of Syrian refugees living in the catchment area. Syrian refugees attending ICRC supported health facilities had bigger families, lower educational level, higher unemployment rate, and were more heavily dependent on humanitarian aid for securing their income, compared to Syrian refugees in the same areas. Among the host Lebanese communities, those attending ICRC supported health facilities were less educated and reported lower access to employment opportunities than the general population (Table 2).

### Perceived key health needs and utilization of primary health care services

In the household survey population, 44.07% (95% CI 41.24–46.9) had sought care within the last month: 49.90% (95% CI 45.42–54.38) of Syrian refugees and 40.09% (36.46–43.72) of Lebanese respondents (*p* value < 0.001). Among the Lebanese respondents, 26.15% (95% CI 23.85–28.45) reported that they had never sought care or that it had been more than a year since their last visit to a health facility. Syrian refugees were more likely to seek care, and more likely to seek it recently (*p*-value 0.04), but were also more likely to have not received care after seeking it (p-value < 0.001). Lebanese participants were most likely to have sought care at a private clinic in their last visit (31.77, 95% CI 28.10–35.44), while Syrian refugees were most likely to have attended another primary health care center (26.70, 95% CI 22.56–30.84), although the differences did not achieve statistical significance.

In the household survey, non-communicable diseases (NCDS) and sexual and reproductive health (SRH) issues were reported as the most common reasons for visit (40.58 and 28.57% respectively). The most common chronic conditions reported were, in order of frequency, arthritis and/or other musculoskeletal conditions, hypertension and diabetes. In contrast, within the clinic population, child health care (such as vaccinations, fever, acute respiratory infection, and diarrhea) was reported as the most common reason for the current health care visit (Table 3).

**Table 3:** Reasons for visit among respondents to the household and clinic surveys (Reasons for visit that did not fall into these categories excluded)

	Household Survey	Clinic Survey
SRH n (%)	276	23
	(28.57)	(16.43)
NCDs n (%)	392	26
	(40.58)	(18.57)
Child Health n (%)	215	73
	(22.26)	(52.14)
Dental Care n (%)	83	18
	(8.59)	(12.86)

Respondents to the household survey indicated preferences for certain types of health centers, depending on the reason for their health visit. Overall, the ICRC-supported clinics were the most popular option for child health ailments (37.84% of care seekers for acute respiratory illness, ARI) and for NCDs (30.30 and 22.95% of diabetes and high blood pressure cases, respectively), while private clinics were frequently chosen for dental care (35.44%), pregnancy related concerns (19.69%), and gynecological problems (36.72%).

For antenatal care (ANC) services, approximately a third of women in the household survey reported no ANC visits in their last pregnancy (32.75, 95% CI 29.00–36.50): For Lebanese respondents 36.82% (95% CI 31.33–42.31) and 29.74% (95% CI 24.65–34.83) for Syrian refugee respondents. This difference was marginally statistically significant – *p*-value 0.05 (Table 4). In the clinic survey, ANC coverage was reportedly high in all locations.

**Table 4:** ANC attendance and relative measures of frequency in the surveyed populations

		Percentage with ANC visits	Mean # of visits	Median # of visits	Range
Household Survey	Lebanese	(63.18)	6.66	7	1–15
	Syrian refugee	(70.26)	5.31	4	1–15
Clinic Survey	Lebanese	(87.5)	4	4	4–6
	Syrian refugee	(98.96)	4.24	4	1–9

Among household survey respondents who had delivered a baby in the last 2 years, only a small percentage of Syrian (4.71, 95% CI 1.71–7.71) and Lebanese women (1.12, 95% CI 0–2.65) had delivered at home. The clinic survey results confirmed a high prevalence of hospital delivery (96.63% of Lebanese women; 86.91% of Syrian refugee women). No Lebanese women and only three out of 91 Syrian refugee women who gave birth in the last 2 years had done so at home.

In terms of utilization of family planning services, 41.73% (95% CI 38.61–44.85) of the respondents to the household survey reported being able to obtain these services whenever needed, with no statistical differences between Syrian and Lebanese respondents. Among respondents who sought family planning services, the greatest percentage did so at private clinics (23.13, 95% CI 13.04–33.22). Lebanese women, however, were statistically less likely to obtain contraception than Syrian refugees: 54.11% (95% CI 46.68–60.54) of the Lebanese respondents reported not receiving it, compared to 43.63% (95% CI 36.06–51.20) among Syrian refugees (*p*-value 0.04).

In the household survey, self-reported vaccination completion did not vary significantly among locations. In Nabatieh, 65.82% (95% CI 58.43–73.21) of respondents reported that their children were on track for their vaccinations, similar to reports from the Beqaa (67.85, 95% CI 59.70–76.00) and Akkar (72.96, 95% CI 65.47–80.45).

For breastfeeding counseling, Lebanese participants were significantly less likely to have breastfed their children than were Syrian refugees: 18.97% (95% CI 15.85–22.09) said they did not breastfeed, compared to 10.71% (95% CI 7.85–13.57) among Syrian refugees (*p*-value < 0.001).

For dental care, despite the high prevalence of expressed dental health needs, 62.00% (95% CI 57.65–66.35) of Syrian refugees reported that they could not visit the dentist when needed, compared to 21.11% (95% CI 18.09–24.13) of Lebanese (*p*-value < 0.001).

### Barriers to utilization of health care services

Among the respondents to the household survey, lack of awareness was cited as the main reason for not attending the ICRC supported facility (54.22% of respondents reported that they were not aware of the ICRC supported clinic services). The difference between Lebanese and Syrian refugee populations was not significant (p-value 0.067).

The majority of Lebanese and Syrian refugees in the household survey population reported that they were paying out of pocket for their health services (Table 5). Compared to Lebanese respondents, Syrian refugees were significantly more likely to report having paid out of pocket for their last health care visit (74.2% versus 80.9% respectively, p-value 0.01). Lebanese respondents reported a higher average expenditure out of pocket per episode of sickness compared to Syrian refugees (104 USD versus 68 USD respectively), regardless of the type of facility attended. The average cost of delivery was similarly higher for Lebanese women (278 USD) compared to Syrian refugee women (190 USD).

**Table 5:** Average out of pocket expenditure during the last episode of care seeking among the surveyed populations

		Lebanese	Syrian refugee
Household Survey	Percentage Paying Out of Pocket Last Visit	74.16%	80.86%
	Out of Pocket Last Visit (USD)	104.35	68.47
	Cost of Delivery (USD)	278.51	190.27
Clinic Survey	Percentage Paying Out of Pocket Last Visit	45.83%	40.68%
	Out of Pocket Last Visit (USD)	2.56	4.08
	Cost of Delivery (USD)	88.00	183.26

In the household survey, the main reasons respondents reported for difficulties in obtaining their prescribed medications were either lack of availability of the prescribed treatment at the facility level (40.27% overall, 95% CI 31.23–49.31) or cost of the treatment (51.77% overall, 95% CI 42.56–60.98).

## Discussion

The study described here originated in the need to understand whether the ICRC support to health facilities was contributing to improve access to preventive and curative services for those it intended to reach (the populations most heavily affected by the Syrian crisis in Lebanon) including both registered and unregistered Syrian refugees along with vulnerable Lebanese.

The results of this study show that the ICRC PHC program in Lebanon has achieved its primary goal of reaching some of the most vulnerable populations (Syrian and Lebanese) in underserved catchment areas of supported facilities. This complex cross-sectional research design also allowed us to confirm that poor Lebanese are much affected by the Syrian crisis but not necessarily reaching supported facilities. Combined, these results show that it is very difficult in a protracted crisis to “leave no one behind” [9–11].

This study, unlike many others, managed to represent catchment areas in the North, the Beqaa as well as the South, a reach that has proved to be very difficult to accomplish in previous studies where sensitive areas were inaccessible [12–14]. The ICRC PHC support program in Lebanon has been implemented mainly along the border areas between Lebanon and Syria, areas that have for decades been inhabited by Syrian seasonal workers and have traditionally been defined as “underserved” [15]. Once the crisis erupted, these same areas, where commercial and social exchanges had long been taking place, became the natural choice for where the Syrians fleeing violence would settle with their families, competing with poor Lebanese for access to basic services including health care. Nine years after the onset of the conflict in Syria, these underserved areas host tens of thousands of Syrian refugees living in difficult circumstances, intermingled with poor Lebanese populations who were having to adjust to more complex social conditions and decreasing employment opportunities within the overall Lebanese economy [3, 16].

Several articles in a recent series on health in humanitarian crises have called for better evidence of effectiveness to inform health programming in crisis settings and have stressed the need for more high-quality research based on assessment of population-based outcomes [17–19].

Other recent works highlight the gaps in terms of quality, quantity, and type of populations covered in research in humanitarian settings [17, 20]. Among populations and topic areas most often neglected, a recent literature review found very little research in particular on SRH in the Middle East [17]. In this study, the research team identified such gaps at the facility level and further sought to understand whether these gaps reflected as well substantial gaps at population level [7]**.**

Reported gaps in the household survey were found in the delivery of SRH and NCD services, regardless of the number of facilities or actors providing these health services. The survey results reveal even higher unmet needs in terms of family planning and ANC services than what has been reported in other studies conducted in Lebanon [21–24]. Such discrepancies might be explained in terms of our use of representative sampling methodology, allowing us to capture the health seeking behaviors among unregistered Syrian refugees, as well as those of registered Syrian refugees. In many other studies, the sampling frame for the Syrian refugee population has been the UNHCR register [4, 8], convenience sampling [16], or a sampling calculation based on UNHCR register [14].

Although SRH care was an important expressed need, the most common reason reported in the household survey as to why women of reproductive age came to health facilities was related to NCDs, which altogether constituted around 40% of the reasons for consultation. While the burden of these conditions in the general population living in Lebanon is expected and corroborated by other studies conducted in the country [12], it was not expected to find that women of reproductive age are more affected by chronic conditions than they are by SRH concerns. This aspect has not yet emerged in other studies conducted among this specific demographic group. Considering the overwhelming evidence on the links between pregnancy outcomes and the future risks that NCDs pose for both the mother and her offspring [25–27], this observation merits further attention in terms of response and more in-depth research.

In terms of child health, caretakers reported low utilization of key preventative health care services such as immunization. Although this study was not designed or powered to estimate vaccine coverage, the findings are consistent with what has already been reported in Lebanon and other countries in the Middle East hosting Syrian refugees [28, 29].

Dental care is another important gap identified that is not supported sufficiently. While poor oral health is a well-documented indicator and risk factor for negative health events, both related to adverse pregnancy outcomes [30] and to the increased risk of NCDs [31, 32], negligible attention has been given to granting access to it in protracted humanitarian crises.

In the household survey, lack of awareness of service availability emerged as a key barrier for utilization of ICRC supported facilities. This finding confirms what was also observed in other surveys investigating access to and utilization of health care services in Lebanon, including the decreasing awareness of Syrian refugees of primary health care services available [8]. Despite the call from UNHCR to strengthen outreach to refugees in order to increase their knowledge of available health services in Lebanon, the centrality of this specific population-level focus receives little attention in the existing humanitarian literature on utilization of health care services in the country. Yet without such outreach as initiated in this study at the population level, it is not possible to design adequate humanitarian programs for implementation, monitoring, and documentation. Routine facility-level monitoring provides insufficient understanding of population needs.

Affordability of health care is another factor identified in previous reports and publications [3, 8, 12, 14, 24]. This study confirms the prohibitively high cost of certain key health care services not only for Syrian refugees, but for uninsured Lebanese as well. It highlights the need both to control costs and to ensure sustained financial support for the most vulnerable Lebanese and Syrians.

The findings of this study allowed the ICRC to identify shortcomings and areas for improvement to be addressed in program implementation at the facility and community level.

The ICRC has highlighted in recent years the need for new evidence-based, long term approaches to the global humanitarian response in protracted crisis [33]. Conducting research in humanitarian crises can be complex: humanitarian settings impose their specific limitations in terms of human and financial resources, time, and/or technical capacity [17, 20, 34]. Humanitarian actors are often not trained to perform research and funds need to be prioritized for operations. Conducting research in an operational context also requires the right combination of assets at the right time [35, 36]. It is also ethically delicate to conduct research in the midst of a crisis [37], since the cost and burden of research cannot be at the expense of the beneficiaries and the questions it intends to answer must be shown to have operational relevance [36].

Conducting research in humanitarian settings remains necessary to ensure that the responses implemented are appropriate for the most vulnerable [38] and reach all of them, which is sometimes not the case [11]. Partnerships with academic institutions can strengthen humanitarian and national health actors in formulating their field knowledge to address some of the shortcomings mentioned above and to generate the much needed evidence for improving humanitarian health programming and delivery [36, 39].

In terms of programmatic responses, a stronger link is required among humanitarian health interventions, the health system, and targeted communities in order to promote awareness and build trust between care providers and service users [40]. Addressing the cost barrier is more complex and caution must be taken to avoid perverse incentives.

This research team in close collaboration with national health providers intends to use the results to readjust the response, knowing that in protracted crises humanitarians must swiftly meet emergency needs while supporting the host country system in shaping strategies to support longer-term complexities [33]. The hard task is to be able to be “working short and long” [33] to support the overall resilience of the systems and people [41].

### Limitations

This study was powered to identify the population attending the ICRC-supported facilities in order to establish whether or not the most vulnerable women and children in the population reached ICRC supported facilities. Although not powered to detect statistically significant results in terms of intermediate health outcomes, the findings are consistent with what is described in similar studies conducted in Lebanon, and therefore we would argue that they carry a certain degree of validity.

We cannot exclude a marginal risk of selection bias, mainly due to the fact that this study did not collect data on reasons for non-response among the eligible participants who declined to participate. We deliberately avoided collecting information on non-responders due to sensitivities around data collection in the specific target areas of the study.

Observer bias could have been introduced by the different background of interviewers in the different locations: In Nabatieh and Akkar this study employed Lebanese Red Cross (LRC) volunteers; in the Beqaa a mix of LRC volunteers, non-LRC, Syrian and Lebanese conducted the interviews. Potential differences might have occurred in the interviewing methodologies adopted by Syrian and Lebanese interviewers in relation to the nationality of the respondents. The study attempted to minimize such risk to the best possible extent through standardized training and constant field supervision from ICRC health field officers.

Despite the homogeneity in terms of socio-demographic profiles of the two study populations, we cannot rule out the possibility of some confounding variable not assessed in this study, since socio-economic dimensions are difficult to capture comprehensively.

In the clinic survey, vulnerable Lebanese are underrepresented. This result may be due to inherent preference among the Lebanese for private facilities, as already described for this population [7, 13, 14]. It is also possible that over the years of this crisis the Lebanese population has preferentially sought out health facilities affiliated with different sponsors or decided to go to clinics that offered a different array of health care services [42]. Other possibilities, including the burden of care on the facilities due to the presence of refugees, the longer waiting time, and consequently the shorter time dedicated to patients’ care, require further exploration. The research team recognizes that the ICRC strategy must include Lebanese hosts more proactively and must strengthen links between the community and the service providers.

## Conclusions

This study identified important gaps in terms of utilization of health care services for women of reproductive age and children, particularly in immunization, SRH and NCD care, despite the support and availability of such services at facility level. It also casts light on oral health as a neglected health need in humanitarian settings. These findings confirm the need to anchor interventions in the community and develop links with existing facilities to which patients can relate and trust.

The study findings also suggest that a stronger community outreach effort is needed to improve awareness of availability of services and knowledge about the importance of preventive care. Support to services provision must also ensure that adequate human resources are available and essential medication proactively prescribed by health care providers to avoid preventable out-of-pocket spending.

The findings of this study highlight the importance of integrating research in the early stages of the humanitarian effort to ensure that important gaps in hard-to-reach populations are identified and responded to. The insights gathered here serve to provide the ICRC and others with important information regarding necessary adjustments and improvements in current health services provision to vulnerable populations in Lebanon. In protracted conflicts, it is necessary to undertake systematic integration of population-based tools to integrate those that are unaccounted for.

The knowledge gained from such inquiry, as the experience of this study shows, then helps to direct the next phase of longer-term and more ambitious efforts to improve health status of conflict-affected populations.

## Abbreviations

ANC: Antenatal Care
CAS: Central Administration of Statistics
CI: Confidence interval
FGDs: Focus Group Discussions
FXB: François-Xavier Bagnoud
ICRC: International Committee of the Red Cross
IRB: Institutional Review Board
ITSs: Informal Tented Settlements
LRC: Lebanese Red Cross
MoPH: Ministry of Public Health
MoSA: Ministry of Social Affairs
NCDs: Non-Communicable Diseases
NGOs: Non-Governmental Organizations
PHC: Primary Health Care
SRH: Sexual and Reproductive Health
UNHCR: United Nations High Commissioner for Refugees
UNICEF: United Nations Children Fund
WHO: World Health Organization
